# Economic Evaluations of Health Service Interventions Targeting Patients with Multimorbidities: A Scoping Literature Review

**DOI:** 10.5334/ijic.8623

**Published:** 2025-01-23

**Authors:** Lucia Ferrara, Vittoria Ardito, Valeria D. Tozzi, Rosanna Tarricone

**Affiliations:** 1 Center for Research on Health and Social Care Management (CERGAS), SDA Bocconi School of Management, Milan, Italy; 2 Department of Social and Political Sciences, Bocconi University, Milan, Italy

**Keywords:** economic evaluation, health service interventions, multimorbidity, chronic conditions

## Abstract

**Introduction:**

Multimorbid patients have been growing, leading to an exponential increase in healthcare costs and patterns of resource utilization. Despite the heightened interest toward integrated care programs as a response to the complex need of multimorbid patients, economic evaluations of these programs remain scarce. This work investigated the economic evaluations of service interventions targeting multimorbid patients, to identify the characteristics of these programs and the methods applied to their evaluation.

**Methods:**

We conducted a scoping review of papers published between 2010 and 2021 on PubMed, Science Direct, EconLit and Web Of Science. The search strategy was built around three keyword blocks: service interventions, multimorbidity, economic evaluations. We selected economic evaluations of service interventions delivered through multiple care settings and targeting patients with 2+ chronic conditions.

**Results:**

Twenty-five articles were included. Interventions were categorized as organizational-type versus patient-oriented. The selected studies often targeted patients with one chronic disease, associated with a mental disorder, like depression or anxiety. Included studies were mostly cost-utility analyses conducted with the healthcare perspective.

**Discussions and conclusions:**

This work confirmed that economic evaluations of service interventions for multimorbid patients are limited in number. This could suggest that decision-making regarding the delivery of healthcare services for multimorbid patients may not always be based on a solid evidence base. More economic analyses are needed to inform evidence-based coverage decision-making.

## Introduction

### Background

The escalating prevalence of multimorbidity is unsurprising, posing a significant challenge to the healthcare systems. As people live longer and advancements in life sciences lead to better health outcomes [1], the incidence of chronic conditions increases with age. Among those aged 65 to 84, and particularly in older populations, the proportion of patients with multimorbidity is constantly rising, standing at 65% and 81%, respectively [2]. This surge in multiple diseases or conditions [3] has a substantial impact on budgets and the sustainability of healthcare systems [4,5] and translates into a considerable increase in healthcare costs and patterns of resource utilization across various resource types, including medications [6,7], primary care and outpatient services [8,9], emergency department access, and hospitalizations [10]. Patients with multimorbidities have complex and intricate needs that span from health to social care, presenting a challenging obstacle for the health systems, necessitating innovative, patient-centred, proactive, and well-coordinated integrated care approaches [11]. Developing such approaches is considered as a mean to achieve the twofold objective of improving care for vulnerable patients and optimizing healthcare resource utilization not only from a patient’s perspective but also from a provider and societal perspective [12].

Despite the heightened interest toward integrated care programs for chronic and multimorbid patients and their growing implementation, recent studies show that economic evaluations of these programs remain scarce [12]. This is attributed both to the specificity of evaluating intervention for multimorbid patients and the challenges associated with conducting economic evaluations of such interventions. Regarding the first point, multimorbid patients present more heterogeneous and complex health profiles, are more likely to be on multiple medications (which increases the risk of drug interactions, side effects, and adherence challenges), and often require coordinated care from multiple specialists and healthcare providers. Each patient’s health trajectory may also be different due to the interplay between various chronic diseases. Evaluating interventions in this context requires careful consideration of the intervention itself, the professionals and resources involved, the complexity of care coordination, more comprehensive disease progression models that consider a broader spectrum of concurrent conditions, and more inclusive outcome measures. These measures should not only focus on disease-specific outcomes, but also on quality of life, functional status, mental health, and patient satisfaction.

Economic evaluations analyses are used to compare alternative health technologies by considering both their costs and associated outcomes. Common methods in economic evaluations include cost-effectiveness analysis (CEA), cost-utility analysis (CUA), cost-consequence analysis, and, occasionally, cost-benefit analysis (CBA) [13]. Economic evaluations are, usually, conducted as part of larger health technology assessments, a multidisciplinary process intended to «determine the value of a health technology at different points in its lifecycle… to inform decision-making (for) an equitable, efficient, and high-quality health system» [14]. While methods for conducting economic evaluations of drugs and medical devices are well established, to date economic evaluations focused on health services interventions, and integrated care programs for chronic and multimorbid patients have not received comparable attention [15,16,17]. Published economic evaluations predominantly focus on specific components of integrated care, and do not fully estimate the overall impact of integrated care projects [17,18].

The complexity of evaluating such interventions arises from the fact that integrated care is a complex package of interventions with varying definitions, compositions, and applications, which substantially deviates from “simple” technologies that are traditionally subject to health economic analysis [17,19]. For such interventions, the adaptation or extension of current economic evaluations methods is required, to address the specificities associated to integrated care [20]. This is because the costs and outcomes span multiple conditions, treatments, and healthcare services (e.g., hospitalizations, outpatient care, home-care medications) making it difficult to attribute costs and resource utilization to specific diseases.

The aim of this paper is to conduct a scoping review of recent economic evaluations of service interventions for multimorbid patients, addressing two research questions:

– What are the characteristics of health service interventions for multimorbid patients in terms of healthcare professionals (HCPs) involved, intervention focus, care setting and outcome measured?
– What economic evaluations are currently conducted to assess such programs?

Addressing these gaps is essential for improving care for multimorbid patients and identifying areas for improvement in current evaluation methods.

## Research methods

### Study design

This work was conducted as part of an Horizon 2020 project, Geronte (Streamlined Geriatric and Oncological Evaluation Based On Ic Technology For Holistic Patient-Oriented Healthcare Management For Older Multimorbid Patients), a European Union (EU) initiative whose ambition is to propose a major step-change from a fragmented system of care to a more integrated and coordinated care pathway for older multimorbid patients. It was developed based on the updated methodological guidance for scoping reviews [21], and followed the 22-item PRISMA-ScR checklist [22].

### Search strategy

Relevant articles were searched in four electronic databases, namely Pub Med, Science Direct, Econlit and Web of Science. Studies published between January 2010 and June 2021 were searched. The starting period was set in 2010 because of the increased attention posed by international bodies towards improving quality of care for older chronic people (e.g., the EU resolution on long-term care for older people (2011/C 308 E/13) [23]). Snowballing was used to identify other relevant studies.

The search strategy was developed around three content blocks (health service interventions, multimorbidity, economic evaluations), and performed on title/abstract. The results were imported into Zotero [24].

### Selection criteria

Full or partial economic evaluations of health service interventions for multimorbid patients were searched. Multimorbidity was defined as the simultaneous presence of two or more disease conditions [25]. To reflect the complexity of care delivery for multimorbid patients, only economic evaluations of clearly-identified, structured, multi-dimensional health service interventions delivered by a team of multidisciplinary specialists and in multiple care settings were considered. Both research articles and research protocols were included. Prior reviews were excluded from data synthesis, but their bibliographies were screened to include additional relevant articles. Only studies published in English and set in middle- or high-income countries were selected for data synthesis.

### Study selection

Studies were selected by two primary researchers (V.A., L.F.). Studies were initially screened based on title and abstract. Disagreements over final inclusions based on full-text read were solved by two other reviewers (R.T., V.D.T.).

### Data extraction

Data extraction was performed in Excel. First, general information of the selected studies was retrieved (year and country of publication, study design). Then, details about the health service interventions were retrieved (intervention description, study setting, target population, conditions treated, HCPs involved). Primary and secondary outcomes of the study (and metrics) were also mapped. Lastly, information on the economic evaluations was extracted (full vs. partial, type of analysis, outcome and cost measures, perspective). Table 1 summarizes all methodological aspects.

**Table 1:** Methodological aspects.


STUDY DESIGN	SCOPING REVIEW

*Information sources*	Pub Med, Science Direct, EconLit, Web of Science

*Keyword search*	(service intervention) OR (care pathway) OR (patient journey) OR (care program) *AND* (multimorbidity OR multimorbid OR comorbidity OR comorbid OR (chronic disease) *AND* (economic evaluation) OR methods OR (cost-effectiveness) OR (cost-utility) OR (cost-benefit)

*Time frame*	10+ years (2010–2021 June)

*Inclusion criteria*	Geography: Studies set in medium- and high-income countries Study design: economic evaluations Study type: research articles and protocols Study object: Single service interventions Target population: Adult populations Setting of care: Multiple care settings

*Exclusion criteria*	Geography: Studies set in low-income countries Study design: Literature reviews and meta-analysis Target population: paediatric patients Setting of care: Single settings of care


### Data analysis

First, a descriptive overview of the selected studies was provided. Then, a descriptive overview of the healthcare interventions followed, in terms of typology, target patients, healthcare professionals, care settings and outcome metrics. Specifically, the intervention types were evaluated using the Cochrane Effective Practice and Organisation of Care categorization [26], used by Smith and colleagues [27], that distinguishes organizational-type interventions against patient-oriented interventions. Organizational-type interventions involve organizational changes primarily delivered by healthcare professionals and relates to case management, coordination of care or enhancement of skill mix in multidisciplinary teams. The organizational complexity of health service interventions for chronic, multimorbid patients was also analysed using six dimensions for achieving efficiency gains throughout the entire care pathway, as outlined by Ferrara et al. [28]. These dimensions represent key areas where efforts can be made to improve the care pathway: adoption of process management tools and resources, data utilization, setup of integrated service delivery, use of ICT & technology, human resource management, and financing. Based on this classification, we analysed which dimensions were observed in the organizational-type interventions selected in the review. Patient-oriented interventions, on the other hand, include any intervention directed primarily at individuals, such as psychoeducational programs and individual or group sessions aimed at enhancing patients’ self-management and self-efficacy.

Outcomes were scrutinised according to the Core Outcome Set (COS) taxonomy by Dodd and colleagues [29], that classifies the outcomes in medical research according to five core areas (i.e., death, physiological/clinical, life impact, resource use, adverse events).

Lastly, the characteristics of the economic evaluations of health service interventions currently available were reported. The quality of the economic evaluation was assessed using the Consolidated Health Economic Evaluation Reporting Standards (CHEERS) checklist [30].

As a final step of the scoping review method [21], the preliminary results were discussed with project consortium members (clinical partners, implementation partners, scientific societies, citizen representatives), both to gather inputs on the research question and sources of information, and to provide interpretative insights on the review findings not yet explored in the literature.

## Results

### Overview of the selected studies

**Figure 1:**
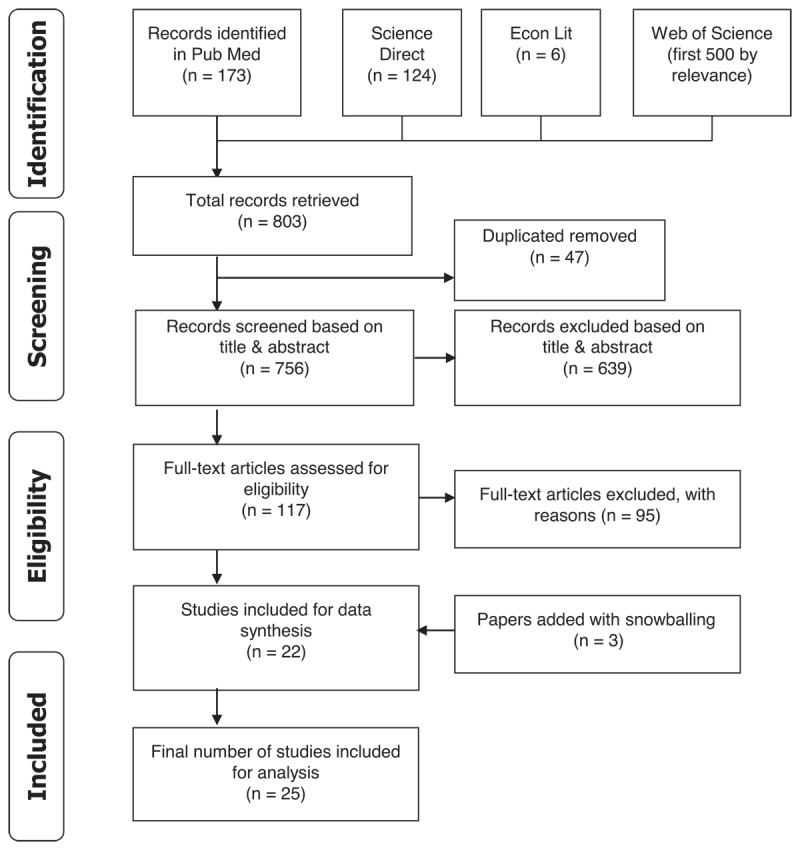
PRISMA flowchart.

A total of 803 records were extracted from the search. After de-duplication, we excluded 47 citations. The remaining papers (n = 756) were screened based on titles and abstracts. Out of these, 639 records were excluded, and the full-text of 117 publications was assessed. Finally, 25 studies were considered for data synthesis (Figure 1).

Selected studies were published between 2011 and 2021, with peaks in 2020. Seven were set in the UK [31,32,33,34,35,36,37], 11 in EU countries (five in Spain [38,39,40,41,42], three in Germany [43,44,45], one in each of Denmark [46], Ireland [47] and the Netherlands [48]), four in Canada [49,50,51,52], two in the United States [53,54], and 1 in Australia [55]. Fourteen studies (56%) were research articles, 11 (44%) protocols. As for study design, most of the studies (N = 22, 88%) were randomized controlled trials (RCT). Table 2 summarizes the study characteristics.

**Table 2:** Summary of the study characteristics.


STUDY CHARACTERISTICS	N (%)

*Publication year*	

2011–2013	7 (28%)

2014–2017	8 (32%)

2018–2021 (June)	10 (40%)

*Country*	

Europe	

UK	7 (28%)

Spain	5 (20%)

Germany	3 (12%)

Other	3 (12%)

North America	

Canada	4 (16%)

US	2 (8%)

Australia	1 (4%)

*Study design*	

RCT	22 (88%)

Quasi-experimental	3 (12%)

*Study type*	

Research article	14 (56%)

Protocol	11 (44%)


### Overview of the health service interventions

#### Type

The interventions illustrated in the selected studies were multifaceted. Using the classification by Smith et al. [29], the interventions were distinguished between organizational-type interventions (about two-thirds) and patient-oriented interventions (one-third). However, while some interventions could unambiguously be categorized as organizational-type or patient-oriented interventions, others presented mixed components [52].

For organizational-type interventions, intervention complexity was assessed using the six-dimension framework for achieving efficiency gain developed by Ferrara et al. (2022) [28]. These interventions include the introduction of process management tool and resources, the use of data and artificial intelligence to support decision-making, the design and delivery of integrated health services around a specific need or population, the use of ICT and technology, human resource management to foster collaboration and workforce redesign, and strategies to reform payment systems to transition toward more value-based reimbursement models. For patient-oriented interventions, several individual dimensions are addressed (i.e., education, self-management and self-efficacy). Supplementary Tables 1 and 2 categorize both organizational-type and patient-oriented interventions, respectively, based on the aforementioned frameworks.

#### Target patients

**Figure 2:**
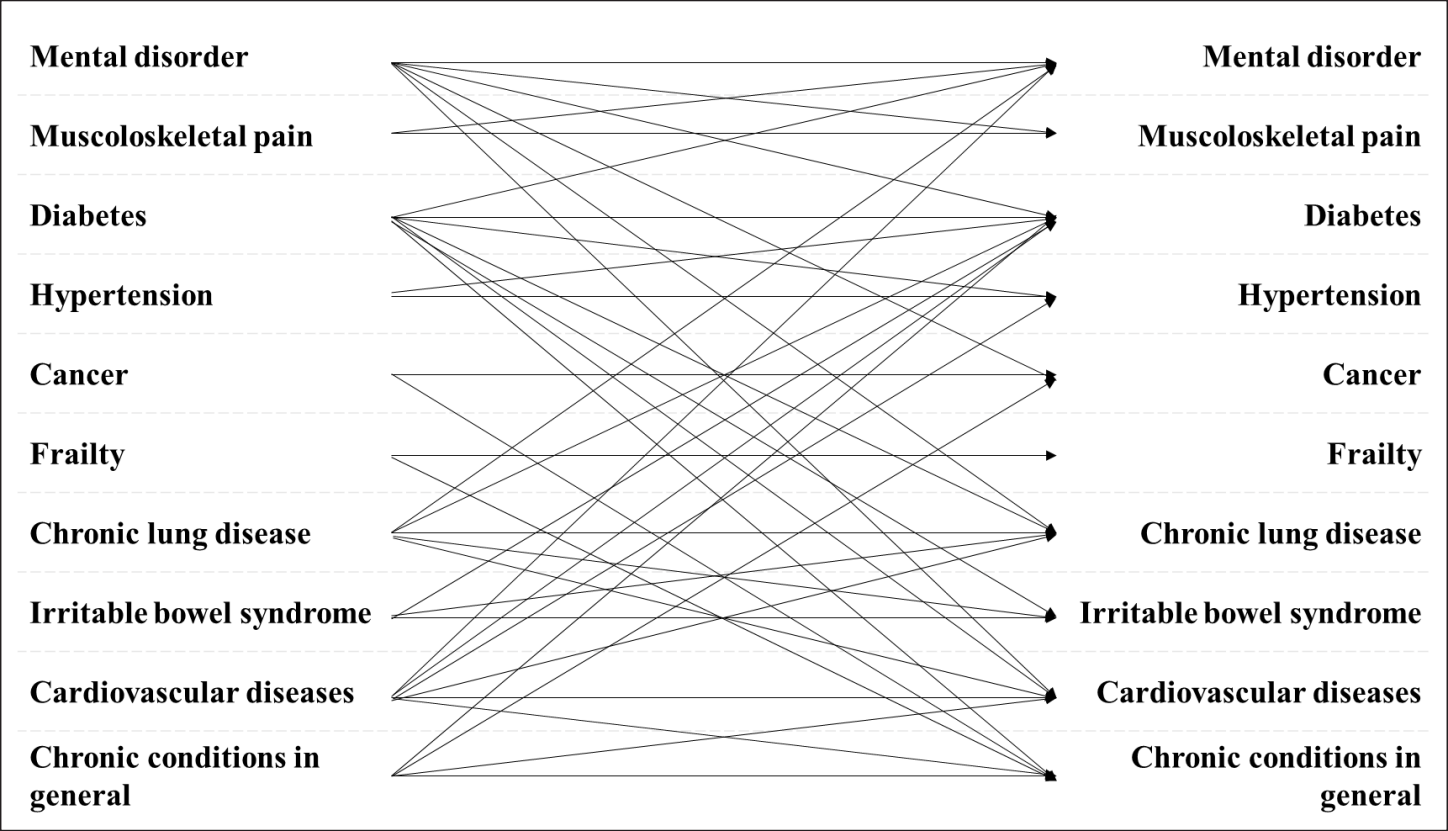
Combination of morbidities in target studies.

The conditions more frequently treated were cardiovascular diseases, including heart failure, ischemic heart disease, or other cardiac conditions (N = 11; 44%), diabetes (N = 10; 40%), and mental disorders (N = 9; 36%). Some health service interventions targeted multimorbid patients in general (N = 11; 44%). Two studies (8%) targeted comorbid cancer patients [32,44]. At an aggregated level, 12 interventions (48%) target two comorbidities, six (24%) three comorbidities, one (4%) six comorbidities, and six (24%) refer to multiple morbidities in general. Figure 2 illustrates the combination of the morbidity profiles.

#### Healthcare professionals

The delivery of health service interventions for multimorbid patients typically involved multiple HCPs. Most interventions involved two (N = 10; 40%) or three (N = 11; 44%) HCPs with different roles. One study refers to HCPs in general, without specifying their specialty and role. The HCPs most represented were general practitioners (GPs) (N = 14; 56%) and nurses (N = 15; 60%). Some nurses were trained by disease-area, especially when conditions required high specialization (e.g., oncology nurse). Four studies (16%) involved specialty doctors, four (16%) pharmacists, and 14 (56%) other professionals, like psychologists, physiotherapists, or dieticians.

Care/case managers act as coordinators of care pathways involving multiple professionals, with their primary focus being defining the necessary steps to recover, + organizing the care plan overall (case manager) [56], or taking care of the patient alongside the care pathway (care manager) [57]. Six of the selected studies (24%) had this profile, in three cases assumed by specialized nurses.

#### Care setting

Healthcare delivery for multimorbid patients often involves various care settings, with almost the totality of the interventions being delivered via two healthcare settings (N = 23; 92%). The analysis focused on assessing the combinations of different care settings. Primary care emerged as the most common care setting (N = 17; 68%), followed by hospitals (N = 7; 28%) and home care (N = 7; 28%), and community setting (N = 5; 20%). Digital components were observed virtually in every selected study, and were either a core channel to deliver the intervention (e.g., platforms for symptom monitoring or Apps for real-time patient-physician communication), or a facilitator (e.g., unstructured or not formalized use of the telephone to follow-up with the patient).

#### Outcome metrics

Table 3 reports core areas based on the Dodd taxonomy [29], the top three outcome domains for each core area, and examples of outcome measures for each domain.

**Table 3:** Outcome area, domains and measures.


CORE AREA	# OF OUTCOME OBSERVED	TOP 3 OUTCOME DOMAINS	EXAMPLES OF OUTCOME MEASURES

Life impact	75	Delivery of care	Satisfaction with care; Patient Assessment of Chronic Illness Care

		Perceived health status	Quality of life, via EQ-5D

		Physical functioning	Instrumental Activities of Daily Living scales; Short-Form 12

Resource use	29	Economic	Costs, cost-effectiveness

		Hospital	Hospital admissions, length of stay, emergency care

		Need for further intervention	Medications

Physiological/clinical	28	General outcomes	Blood pressure, cholesterol, changes in levels of clinical parameters

		Psychiatric outcomes	Center for Epidemiologic Studies Depression scale; Hopkins Symptom Checklist; “Depression-free days”

		Other outcome areas	E.g., cardiac outcomes or metabolism and nutrition outcomes

Death	5	Mortality/survival	Mortality, survival

Adverse events	1	Adverse events/effects	Variables of clinical efficacy (e.g., number of episodes of worsening of heart conditions)


“Life impact” stands out as the primary core area where a majority of the outcome metrics were observed. “Delivery of care”, “perceived health status” and “physical functioning” were the domains observed with the higher frequency.

“Physiological/clinical” outcomes followed, related to the disease condition studied. For instance, for interventions targeting mental disorders, psychiatric outcomes were assessed using the Center for Epidemiologic Studies Depression [58] scale or the “Depression-free days” questionnaire [59].

“Resource use” outcomes were also core, including metrics within three domains that correspond to direct healthcare costs as described in Drummond et al. [13]: “hospital”, “economic”, and “need for further intervention”. The “hospital” domain comprises any information on resource use related to hospital services (hospital admissions, length of stay, emergency services). The “economic” domain comprises information on cost and/or cost-effectiveness. The “need for further intervention” domain comprise information on medications (number of drugs, daily doses).

“Death” (i.e., mortality rate, survival, or clinical remission) and “adverse events” (i.e., unexpected worsening of health conditions) were only sporadically observed.

### Economic evaluations of health service interventions

Of the selected studies, 22 were full economic evaluations (comparatively assessing both outcomes and costs of the intervention group and its comparator [13]) (Table 4). Cost-utility analyses (CUA) were the most common type (N = 17, 77%). Two studies (9%) were cost-effectiveness analyses (CEA), and three studies (14%) conducted both a CEA and a CUA. Eleven studies (50%) assumed a healthcare perspective, six studies (27%) a societal perspective, four studies (18%) had the two-fold perspective of both the healthcare system and society. One study (5%) assumed the perspective of the insurance payer.

**Table 4:** Characteristics of full economic evaluations.


AUTHOR	TYPE OF EE	OUTCOME MEASURE	DIRECT HEALTHCARE COSTS	DIRECT NON-HEALTHCARE COSTS	PRODUCTIVITY LOSSES	PERSPECTIVE OF ANALYSIS

Aragones, 2020 [39]	CUA	QALYs, using EQ-5D-3L	Yes	No	Yes	Healthcare; Societal

Belnap, 2019 [53]	CUA	QALYs, using SF-12 scores	Yes	No	No	Health insurance payer

Piera-Jiménez, 2021 [40]	CEA	QALYs, using the Barthel index scale	Yes	Yes	No	Healthcare; Societal

Evans, 2011 [31]	CEA	EQ-5D-5L, Crosswalk Index value	Yes	Yes	No	Not indicated (supposedly, societal)

Duarte, 2015 [32]	CUA	QALYs, using EQ-5D-3L	Yes	No	Yes	Healthcare

Thorn, 2020 [33]	CUA	QALYs, using EQ-5D-5L	Yes	Yes	Yes	NHS and Personal Social Service

Henderson, 2013 [34]	CUA	QALYs, using EQ-5D	No	No	Yes	Healthcare and social services

Maru, 2018 [55]	CUA	QALYs, using EQ-5D-3L; LYs	Yes	No	Yes	Healthcare

Russo, 2012 [54]	CUA	QALYs (using a regression model based on vitals levels)	Yes	No	Yes	Healthcare

Camacho, 2016 [35]	CUA	QALYs, using EQ-5D-5L	Yes	Yes	Yes	Societal

Nobis, 2013 [45]	CEA, CUA	QALYs, using EQ-5D and SF-12	Yes	Yes	No	Societal

Rose, 2015 [43]	CUA	QALYs, using EQ-5D	Yes	No	No	Societal

Casanas, 2019 [41]	CUA	QALYs, using EQ-5D-5L	Yes	No	Yes	Not indicated (supposedly, healthcare)

Pedersen, 2021 [46]	CEA, CUA	EQ-5D-5L	Yes	No	No	Healthcare

Martin-Lesende, 2011 [42]	CUA	QALYs, using EQ-5D	Yes	No	Yes	Healthcare

Bower, 2012 [36]	CUA	QALYs, using EQ-5D	Yes	No	No	Not indicated (supposedly, societal)

Gray, 2016 [50]	CUA	HrQoL, using AQoL-4D	Yes	Yes	Yes	Healthcare; Societal

Herkert, 2020 [48]	CUA	QALYs, using EQ-5D-5L	Yes	No	Yes	Healthcare

Johnson, 2012 [51]	CUA	HrQoL, using SF-12 and EQ-5D	Yes	No	Yes	Healthcare

Man, 2016 [37]	CUA	QALYs, using EQ-5D-5L	Yes	Yes	No	NHS and Personal Social Service; Patient

Lanzeta, 2016 [38]	CUA	QALYs, using EQ-5D	Yes	No	No	Not indicated (supposedly, healthcare)

Gillespie, 2017 [47]	CEA, CUA	QALYs, using EQ-5D	Yes	No	Yes	Healthcare


*Abbreviations*: CEA = Cost-effectiveness analysis; CUA = Cost-utility analysis; EE = Economic evaluation; HrQoL = Health-related quality of life; LYs = Life Years; QALYs = Quality Adjusted Life Years; SF = Short Form.

In terms of outcome measures, Quality-Adjusted Life Years (QALYs), measuring for both length of life (i.e., mortality) and quality of life (i.e., morbidity), were commonly used [60]. Most of the studies derived QALYs from the EQ-5D (EuroQoL) scale (based on five or three dimensions) or the Short-Form 12 survey (assessing how health impacts on everyday life) [61].

Based on Drummond et al. [13], Table 4 breaks down the costs in three classes. *Direct healthcare costs* include costs directly associated to the delivery of a health intervention, ranging from hospitalization to medication, from emergency visits to outpatient visits. These were observed in 21 studies (95%). Within this cluster, costs directly associated to the establishment and implementation of the intervention were observed in 15 studies, and included costs to purchase equipment, to train dedicated personnel, and to supervise and monitor. *Direct non-healthcare costs* comprise all costs paid to access a health service, such as travel costs, dining costs, formal caregiver expenses, and were observed in seven studies (32%). *Productivity losses*, accounting for patients’ or caregivers’ work leave, were observed in six studies (27%).

Information on resource use employed in economic evaluations was informed via different sources. Retrospective studies retrieved available data, like administrative databases, health insurance claims or extractions from the general practitioners’ records. Prospective studies typically relied on patient-administered questionnaires to collect data. As for costs, resources were typically valued using officially listed prices from national or local formularies.

Amongst the partial economic evaluations, one work was an outcome description [44], whereas two works were only cost analyses [49,52].

### Quality assessment

The reporting quality of the economic evaluation studies was assessed by the 28-item CHEERS checklist [30]. Each record was labelled as “fully met”, “partially met”, “not meet”, or “not applicable” (Supplementary Table 3). Overall, the evidence was deemed acceptable, with 57% of the items fully meeting the CHEERS requirements. 20% of the items did not meet the CHEERS standards, while in 10% of the cases these were only partially met. 13% of the items were not relevant and quality could not be assessed (e.g., study protocols).

## Discussion

### Summary of evidence

This work aimed at identifying the economic evaluations published to date that assess health service interventions for multimorbid patients. First, for each selected study, the intervention type, target patients, healthcare professionals, care settings, and outcome metrics associated to the health interventions were first evaluated. The outcome of this analysis highlighted that health service interventions for multi-morbid chronic patients address a variety of intricate needs and serve multiple purposes. This is because these patients are affected by two or more chronic conditions that require constant care over extended periods of time (if not lifetime care). Delivering such interventions is rather complex, as they typically involve at least two healthcare professionals from different medical specialties (typically displaced in different hospitals, outpatient clinics, primary care facilities, or other), and span across more than one setting of care (because patients are treatment over time, differently from acute care one-off interventions). This highlights a high need for coordination of the variety of professionals and settings involved, while also exposing to the risk of high coordination costs. In this context, it is becoming increasingly common to leverage digital tools that aim to facilitate communication and coordination amongst professionals, while also possibly contributing to containing the costs. In addition, the features of the economic evaluations, type of analysis, and perspective assumed, were investigated.

This work elaborates on the fact that service interventions for multimorbid patients have a high level of sophistication in terms of dimensions involved, such as the combination of settings of care and the different healthcare professionals, as also discussed in other papers on the topic [17,18]. When adopted in the clinical practice, such interventions tend to simultaneously impact more organizational components, as they require the re-organization of the care pathways, the arrangement of new forms of communication between healthcare professionals, or the coordination among a variety of care settings. These distinctive features make the economic evaluation of integrated care programs complex.

By investigating the economic evaluations of interventions designed for multimorbid patients, this work confirmed that, despite a growing interest, such analyses are still limited in number. The majority of the published economic evaluations were full economic analyses, and mostly CUAs, confirming academic attention on assessing that new care programs or patient pathways should be extensively assessed with an economic perspective, as opposed to only assessing their clinical effectiveness. Economic evaluations of health service interventions were predominantly conducted with the healthcare perspective, despite the patients and their caregivers have a key role in the management of chronic conditions. Conversely, caregiving costs, be it formal or informal care, were typically neglected in the observed studies.

Overall, the current review suggested that multimorbidity is still assessed as a health status that combines two (or more) disease conditions. This often takes the form of a chronic disease (e.g., COPD) that is associated with a mental disorder (e.g., depression or anxiety). Disease-specific outcome measures are used, as opposed to metrics that allow evaluating the patient health status at large. Patient experience with the intervention as a whole was rarely assessed through multi-dimensional patient-reported outcome and experience measures (i.e., PROMs and PREMs), with patients mostly engaged only through satisfaction questionnaires. While the scarce use of patient outcomes in economic evaluations has been observed and discussed with respect to any health technology, it becomes particularly important for health service interventions for multimorbid patients who are exposed to the healthcare services over time. Although patient experience is increasingly recognized as an integral component of service quality and positive patient experiences are associated with better treatment adherence and outcomes [62], patients’ views on the quality of care are not yet systematically collected.

### Limitations

The current work presents some limitations. First, the heterogeneity of the interventions illustrated in the selected studies, as well as their mixed target populations, limit the possibility to evaluate costs and outcomes of the economic evaluations with a comparative perspective. For this reason, we did not analyse quantitatively the findings of the selected studies, nor were able to perform a meta-analysis [30]. A second limitation is linked to the fact that not all chronic conditions are equally represented in the pool of selected studies. For instance, cancer populations are not well represented in the research, especially in rigorous designs (e.g., RCTs). Results should therefore be interpreted with caution and should not be generalized to any intervention for multimorbid patients. A third limitation of the search strategy used in this study is that certain relevant articles or publications may have been inadvertently overlooked. This could be due to the inherent constraints of the databases searched, the use of specific keywords, or publication bias. However, to mitigate this limitation, we incorporated a triangulation approach by supplementing the literature review with expert interviews. These interviews involved healthcare professionals and key stakeholders, allowing for the inclusion of practical insights and experiences that may not be captured in published articles. Additionally, we discussed the preliminary findings from the literature review with these experts, further ensuring that the results reflect a comprehensive understanding of the current landscape. This mixed-methods approach strengthens the validity of our conclusions by integrating both academic and practical perspectives. Lastly, this work only considered studies published in English as inclusion criteria. Other potentially relevant economic evaluations might have been excluded from our analysis on a language-basis.

## Conclusion

This scoping literature review aimed to build on the available evidence on economic evaluations of health service interventions, and to make a substantial contribution to the literature on integrated care by focusing specifically on multimorbid patients and care delivered in multiple settings. Indeed, the presence of at least two chronic conditions and the development of programs that span among several providers, setting and levels of care were both considered as prerequisite for inclusion in this study. This is because, the study advocates for a unified consideration of the needs of multimorbid patients and recognize limitations in current programs that are often single-disease oriented or a combination of single-disease oriented approaches.

Despite being still relatively limited in number of available studies compared to economic analyses of drugs or medical devices, this work outlines a growing academic interest towards performing economic evaluations of health service interventions and care programs, particularly with respect to chronic conditions. The work also reinforces the need to develop robust evaluation to inform allocation decisions in the field of multimorbidity. Indeed, in a context in which established demographic and epidemiological trends illustrate that the population is ageing and that chronic conditions are increasingly prevalent, the scarcity of economic analyses of service interventions on, and of attention towards, multimorbid patients might be evocative of the fact that decision-making related to the delivery of healthcare services for this target group might not be evidence-based. This is alarming *per se*, but becomes even more critical considering the paradigms of value-based healthcare and evidence-based decision-making, which are widely recognized as guiding principles to ensure that only interventions with a strong evidence of cost-effectiveness are used [63]. The scarcity of economic evaluations highlighted with this work might suggest that not all the services for multimorbid patients are cost-effective, and therefore worth paying. Multimorbid patients absorb an enormous amount of economic resources and have a considerable weight on the national healthcare budgets [12]. Globally, approximately one in three of all adults suffer from multiple chronic conditions [64], with this figure expected to grow dramatically [65]. This will result in an exponential increase in healthcare expenditures associated to each chronic condition, as there will be growing demand for specialty consultations, more visits to emergency departments, and higher rates of hospital admissions. In such scenario, evidence-based decision processes would enable a more efficient allocation of healthcare resources, with beneficial consequences not only for patients, but also for national budgets.

Moreover, this work underscores the need for a methodological and pragmatic reflection on how to analyse the evidence derived from studies that focus on multimorbidity. A first well-discussed challenge relates to collecting relevant cost data, attributing costs and resource utilization to specific diseases [12], and measuring the economic impact at the organization and system levels. A second challenge is connected to the poor generalizability of the economic evaluations performed in the area of service interventions. Both the intervention effectiveness and its implementation success are tied to contextual and organizational factors, and are evaluated accordingly. The study findings of the papers included in this review are never discussed with respect to outer settings, and none of the authors strive to increase the resonance of their research by developing frameworks or drawing recommendations to generalize the takeaways to other contexts. This is mainly due to the strong role played by the environment where they are experimented and implemented, and therefore suggest on one side the need to complement the analysis with more realist evaluation approaches that account for the contextual factors that could have had an impact on the results, and on the other side to complement the discussion with broader implication for other context. Lastly, an analysis of the outcome measures gauged in studies of this review highlighted that, although the delivery of care domain is often monitored [29], the overall patient experience with the intervention is scarcely extensively assessed. Most of the studies still uses plain, typically one-question-only scales that assess the overall satisfaction with the health intervention (e.g., “*On a 1–10 scale, how satisfied are you with this care program?*”). More sophisticated patient-reported experience measures (PREMs) do not seem to be yet systematically measured, nor monitored. Given the increasing recognition of patient experience as a critical element of healthcare quality, and its established link to better adherence to treatment and improved outcomes, greater emphasis should be placed on systematically evaluating patient experiences in future research.

## Additional File

The additional file for this article can be found as follows:

10.5334/ijic.8623.s1 Supplementary File. Supplementary Tables 1 to 3.
